# *FTO* rs9939609 polymorphism is associated with metabolic disturbances and response to HCV therapy in HIV/HCV-coinfected patients

**DOI:** 10.1186/s12916-014-0198-y

**Published:** 2014-11-03

**Authors:** Daniel Pineda-Tenor, Juan Berenguer, María A Jiménez-Sousa, Mónica García–Alvarez, Teresa Aldámiz-Echevarria, Ana Carrero, Sonia Vázquez-Morón, Pilar García-Broncano, Cristina Diez, Francisco Tejerina, María Guzmán-Fulgencio, Salvador Resino

**Affiliations:** Unidad de Infección Viral e Inmunidad, Centro Nacional de Microbiología, Instituto de Salud Carlos III, Carretera Majadahonda- Pozuelo, Km 2.2, 28220 Majadahonda, Madrid Spain; Unidad de Enfermedades Infecciosas/VIH, Hospital General Universitario “Gregorio Marañón”, Madrid, Spain; Instituto de Investigación Sanitaria Gregorio Marañón (IiSGM), Madrid, Spain

**Keywords:** AIDS, Chronic hepatitis C, HCV therapy, Metabolism, Insulin resistance, Obesity, SNPs

## Abstract

**Background:**

The *Fat Mass and Obesity-Associated Protein (FTO)* gene rs9939609 single nucleotide polymorphism (SNP) has been associated with obesity, metabolic syndrome, insulin resistance (IR), and type 2 diabetes mellitus in the general population. The aim of our study was to examine for the first time the association of the rs9939609 polymorphism with metabolic disturbances, liver disease and virologic response to hepatitis C virus (HCV) therapy with pegylated-interferon-alpha plus ribavirin (pegIFNα/RBV) in human immunodeficiency virus (HIV)/HCV coinfected patients.

**Methods:**

We carried out a cross-sectional study in 261 patients, of whom 178 were subsequently treated with pegIFNα/RBV therapy. *FTO* rs9939609 and *IFNL3* rs12980275 polymorphisms were genotyped by GoldenGate®. The main outcomes were: 1) metabolic disturbances: insulin resistance (homeostatic model assessment (HOMA-IR)) and overweight (body mass index (BMI)); 2) liver disease (Metavir score): significant fibrosis (F ≥2) and steatosis (>10% fatty hepatocytes); and 3) virologic response to HCV treatment: sustained virologic response (SVR).

**Results:**

The rs9939609 AA genotype was associated with higher values of BMI (adjusted arithmetic mean ratio (aAMR) = 1.08; 95% confidence interval (95%CI) = 1.03 to 1.14; *P* = 0.002) and HOMA-IR (aAMR = 1.32; 95%CI = 1.03 to 1.69; *P* = 0.027). Patients with an rs9939609 AA genotype had higher likelihoods of achieving values of BMI ≥27.5 kg/m2 (adjusted odds ratio (aOR) = 3.46; 95%CI =1.17 to 10.21; *P* = 0.024), HOMA-IR ≥2.5 (aOR = 2.09; 95%CI = 1.02 to 4.32; *P* = 0.045), significant fibrosis (aOR = 2.34; 95%CI =1.02 to 5.36; *P* = 0.045) and steatosis (aOR = 3.65; 95%CI = 1.29 to 10.36; *P* = 0.015). The rs9939609 AT/AA genotype decreased the likelihood of achieving SVR (aOR = 0.58; 95%CI = 0.34 to 0.99; *P* = 0.044). A decision tree was performed with the genotypes of HCV, *IFNL3* and *FTO*. The incorporation of rs9939609 significantly improves the prediction of SVR (P <0.05). The overall accuracy was 68.2%.

**Conclusions:**

Patients carrying the unfavourable AT/AA genotype of rs9939609 polymorphism had higher odds of metabolic disturbances and a lower likelihood of achieving successful virologic response to HCV therapy.

**Electronic supplementary material:**

The online version of this article (doi:10.1186/s12916-014-0198-y) contains supplementary material, which is available to authorized users.

## Background

The combination antiretroviral therapy (cART) has made human immunodeficiency virus (HIV) infection a chronic manageable disease in high income countries [1]. In this setting, chronic hepatitis C (CHC) has turned into an important comorbidity and a major cause of death in HIV/hepatitis C virus (HCV) coinfected patients [2,3], because HIV infection accelerates the natural history of CHC [4-6]. Moreover, although the published data suggest that cART might be beneficial for HIV/HCV-coinfected patients [4], the interactions among HIV, HCV and cART are also associated with several metabolic disorders [7], including dyslipidaemia, lipodystrophy, steatosis, insulin resistance and type 2 diabetes mellitus [7,8].

For many years, dual therapy with pegylated-interferon-alpha plus ribavirin (pegIFNα/RBV) has been the standard anti-HCV therapy for HIV/HCV-coinfected patients [9], and is still used in combination with new direct-acting antivirals, such as telaprevir or boceprevir [10]. The rate of HCV clearance after pegIFNα/RBV therapy is around 20% to 40% for patients infected with HCV genotype 1 (HCV-GT1) and HCV genotype 4 (HCV-GT4), and 50% to 60% in HCV genotype 2 (HCV-GT2) and HCV genotype 3 (HCV-GT3) patients [11,12]. To date, the best baseline predictors for HCV therapy are HCV genotype, HCV viremia, liver fibrosis and single-nucleotide polymorphisms around the *interferon lambda 3 (IFNL3)* gene, also known as *interleukin 28B* (*IL28B*) [13]. However, an unexplained variability in HCV treatment outcome still remains which suggests that other host genetic factors may play an important role in pegIFNα/ribavirin therapy [14]. Thus, the identification of predictors for HCV therapy might help to ensure an adequate selection of the best candidates and to minimize any undesirable toxicity.

At present, the new direct-acting antivirals (DAAs) are generally administered in combination with pegIFNα/ribavirin, particularly in difficult-to-treat patients infected with GT1/4 [15,16]. Moreover, the new IFNα-free regimens with DAAs in combination with or without ribavirin are being developed for difficult-to-treat patients [17]. However, the potential use of these new DAAs in HIV/HCV coinfected patients is complicated due to the choice of patients to treat, potential for drug-drug interactions and overlapping toxicities between HIV and HCV therapy [16]. In addition, the new DAAs are more expensive and there are serious restrictions on their administration, and in many regions in the world these drugs are inaccessible. In fact, treatment with pegIFNα/ribavirin remains the only option of therapy for many patients throughout the world.

The Fat Mass and Obesity-Associated Protein, also known as FTO, is an alpha-ketoglutarate-dependent dioxygenase. The *FTO* gene plays an important role in the management of energy homeostasis and the regulation of body weight [18]. This gene is located on chromosome 16q12.2 and has nine exons which encode a 2-oxoglutarate-dependent nucleic acid demethylase highly conserved in vertebrates [18]. Single nucleotide polymorphisms (SNPs) that cluster in the first intron of the *FTO* gene were first discovered in a genome-wide association study (GWAS) for type 2 diabetes mellitus [19], and, subsequently, other GWAS reported that the *FTO* polymorphisms were associated with obesity [20]. Evidence from epidemiological and functional studies suggests that *FTO* confers an increased risk of obesity by subtly changing food intake and preference [21]. The *FTO* rs9939609 polymorphism, one of the SNPs that were reported, has been associated with obesity [22], metabolic syndrome [23], insulin resistance [24], type 2 diabetes mellitus [19] and cardiovascular disease [25].

Considering that obesity, insulin resistance and steatosis have been identified as important factors that promote metabolic syndrome progression and failure of HCV therapy in HCV-infected patients [26], the aim of this study was to examine the association of the *FTO* rs9939609 polymorphism with the metabolic disturbances and virologic response to HCV therapy with pegIFNα/RBV in HIV/HCV-coinfected patients.

## Methods

### Patients and study design

We carried out a cross-sectional study in 261 HIV/HCV-coinfected patients from Hospital Gregorio Marañón (Madrid, Spain) between September 2000 and July 2009. In addition, we performed a retrospective study in 178 of them who started HCV treatment.

All subjects included in our study were European white and HCV treatment-naive patients, who were potential candidates for HCV therapy and, in most cases, underwent a liver biopsy. The inclusion criteria were: detectable HCV-RNA by polymerase chain reaction (PCR), negative hepatitis B surface antigen, availability of DNA sample, no clinical evidence of hepatic decompensation, no diabetes mellitus and stable cART or no need for cART. Patients with active opportunistic infections, active drug and/or alcohol addiction and other concomitant severe diseases were excluded.

**Figure 1:**
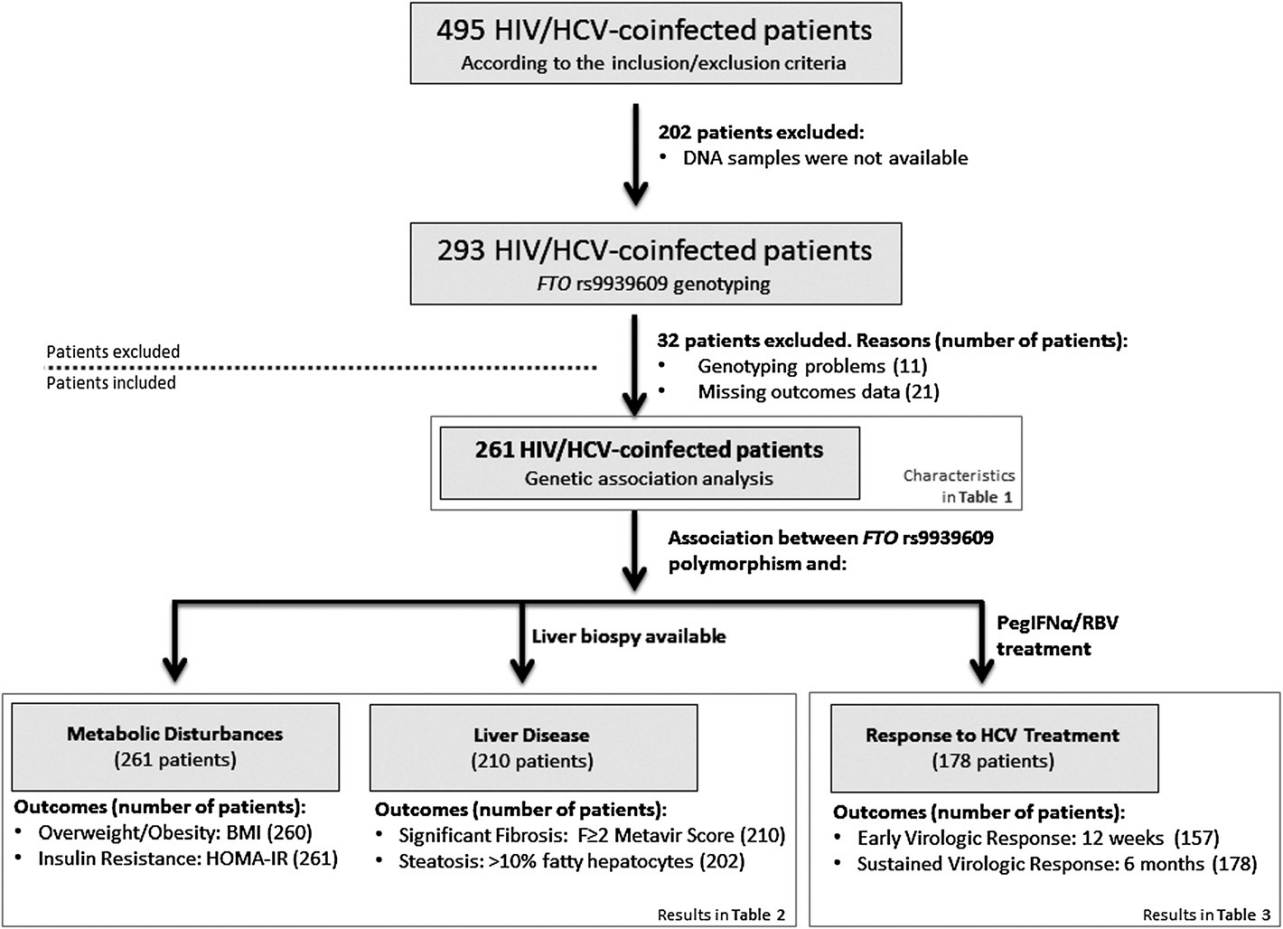
**Flow chart of patients included in the genetic association analysis.** BMI, body mass index; HCV, hepatitis C virus; HIV, human immunodeficiency virus; HOMA-IR, homeostatic model assessment-insulin resistance; pegIFNα/RBV, pegylated-interferon-alpha plus ribavirin.

A total of 495 HIV/HCV-coinfected patients met the inclusion criteria. Of these, DNA samples were available for 293 patients, but only 261 patients were available for statistical analysis: 11 patients were excluded due to DNA genotyping errors (low quantity or quality of DNA, human and technical errors, and so on) or having missing values. In addition, 21 patients were excluded due to missing outcomes data. A total of 210 patients had liver biopsy data and 178 patients were subsequently treated with pegIFNα/RBV therapy (Figure 1).

The study was approved by the Institutional Review Board and the Research Ethics Committee of the Instituto de Salud Carlos III (ISCIII). This study was conducted in accordance with the Declaration of Helsinki and patients gave their written informed consent for the study.

### Epidemiological and clinical data

Epidemiological and clinical data were obtained from medical records. Body mass index (BMI) was calculated as the weight in kilograms divided by the square of the height in meters. The duration of HCV infection for patients with a history of intravenous drug use (IDU) was estimated starting from the first year they shared needles and other injection paraphernalia, which are the most relevant risk practices for HCV transmission [27]. For non-IDU patients, we only included those patients for whom the initiation of their HCV infection could be determined with certainty. The degree of insulin resistance was estimated for each patient using the homeostatic model assessment (HOMA-IR) score described by Matthews *et al*. [28]: fasting glucose (mmol/l) times fasting insulin (mU/l) divided by 22.5.

### HCV assays

HCV infection was documented in all patients by enzyme-linked immunosorbent assay (ELISA) and PCR test. The HCV genotype was determined by hybridisation of biotin-labeled PCR products to oligonucleotide probes bound to nitrocellulose membrane strips (INNO-LiPA HCV II, Innogenetics, Ghent, Belgium). Plasma HCV-RNA viral load was measured by PCR (Cobas Amplicor HCV Monitor Test, Branchburg, NJ, USA) and real-time PCR (COBAS AmpliPrep/COBAS TaqMan HCV test); results were reported in terms of international units per milliliter (IU/mL), with a lower limit of detection of 10 IU/mL.

### Liver biopsy

Liver biopsies were performed on 210 of 261 patients following the recommendations of the Patient Care Committee of the American Gastroenterological Association [29]. Liver fibrosis was estimated according to the Metavir score as follows: F0, no fibrosis; F1, portal fibrosis; F2, periportal fibrosis or rare portal-portal septa; F3, fibrous septa with architectural distortion; no obvious cirrhosis (bridging fibrosis); and F4, definite cirrhosis. Liver steatosis was evaluated according to the existence of hepatocytes containing visible macrovesicular fat droplets. We considered hepatic steatosis to be clinically significant when fatty hepatocytes exceeded 10% of the hepatic parenchyma.

### Hepatitis C therapy

HCV treatment was administered to 178 of the 261 patients. Treatment regimens included pegIFNα 2a or 2b at standard doses (180 μg/week or 1.5 μg/kg/week, respectively) plus weight-adjusted ribavirin dosing (1,000 mg/day for patients weighing <75 kg and 1,200 mg/day for patients weighing ≥75 kg). Following international guidelines [30], patients with HCV genotypes 1 or 4 received either 48 or 72 weeks of treatment, and patients with HCV genotype 3 were treated for 24 or 48 weeks. Early stopping rules were applied for subjects with suboptimal virologic response at week 12.

### Genotyping of DNA polymorphisms

Genomic DNA was extracted from peripheral blood with a Qiagen kit (QIAamp DNA Blood Midi/Maxi; Qiagen, Hilden, Germany). The *FTO* rs9939609 polymorphism and the *IFNL3* rs12980275 polymorphism were genotyped at the Spanish National Genotyping Center (CeGen; [31]) by using the GoldenGate® assay with VeraCode® Technology (Illumina Inc. San Diego, CA, USA).

### Outcome variables

The outcome variables were: 1) metabolic disturbances: overweight/obesity (BMI values and BMI ≥27.5 kg/m^2^) and insulin resistance (HOMA-IR values and HOMA-IR ≥2.5); 2) liver disease: significant fibrosis (F ≥2) and steatosis (>10% fatty hepatocytes); and 3) virologic response to HCV treatment: sustained virologic response (SVR) was a non-detectable HCV viral load (<10 IU/mL) six months after treatment cessation.

### Statistical analysis

All statistical tests were performed with the Statistical Package for the Social Sciences (SPSS) 19.0 software (IBM Corp., Chicago, IL, USA). All *P*-values were two-tailed and statistical significance was defined as *P* <0.05.

For the description of the study population, *P*-values were estimated with a linear regression for continuous variables and a Chi-square test for categorical variables. Continuous variables were expressed as median (interquartile range) and categorical variables as percentage (absolute frequency). Hardy-Weinberg equilibrium (HWE) was assessed by a Chi-square test, considering equilibrium when *P* >0.05.

The genetic analysis was carried out according to recessive and additive genetic models, but we showed the model that best fitted the outcome variable analysed in each case. For the genetic association study, univariate and multivariate generalised linear models (GLM) with normal distribution (log-link) were used to study the association between *FTO* polymorphism and continuous outcome variables (HOMA-IR and BMI). This test gives the differences between groups and the arithmetic mean ratio (AMR) and 95% confidence interval (95%CI). GLM with binomial distribution (logit-link) was used to investigate the association between *FTO* polymorphism and categorical outcome variables (HOMA-IR and BMI cut-offs, liver steatosis and virologic response to HCV therapy). This test gives the differences between groups and the odds ratio (OR) and 95%CI. Each GLM test was adjusted by the most significant co-variables associated with each one of the outcome variables, avoiding over-fitting of the regression. We included the SNP (Enter algorithm) and the most relevant characteristics (Stepwise algorithm. At each step, factors are considered for removal or entry: a *P*-value for entry and exit of 0.15 and 0.20, respectively). The covariables used were gender, age, BMI, AIDS, nadir CD4+ T-cells, undetectable HIV viral load (<50 copies/mL), time on cART, HCV genotype, HCV viral load ≥500,000 IU/ml, HOMA-IR, liver fibrosis and *IFNL3* rs12980275 polymorphism. The adjusted model also takes into consideration the combination of specific antiretroviral drugs used by each patient, including zidovudine, stavudine, didanosine, tenofovir, abacavir, efavirenz, ritonavir, lopinavir, saquinavir and fosamprenavir.

Moreover, decision tree analyses were performed via a classification and regression tree (CART) algorithm to classify patients according to SVR using *IFNL3* and *FTO* genotypes. This analysis provides a prognostic system with a hierarchical structure based on recursive portioning that builds a decision tree to identify subgroups at higher odds of SVR. The accuracy was evaluated by calculating the area under the receiver operating characteristic curves (AUROC). The branches were pruned when groups had fewer than 10 patients.

## Results

### Patient characteristics

Table 1 shows the epidemiological and clinical characteristics of 261 non-diabetic HIV/HCV-coinfected patients. Note that patient’s characteristics were similar when they were stratified by *FTO* rs9939609 genotypes (TT, AT and AA).

**Table 1:** **Clinical and epidemiological characteristics of all HIV/HCV-coinfected patients stratified by** ***FTO*** **genotype**

**Characteristics**	**All patients (number = 261)**	**TT (number = 101)**	**AT (number = 123)**	**AA (number = 37)**	***P*** **-value**
Gender (male)	74.7% (195)	73.3% (74)	74% (91)	81.1% (30)	0.625
Age (years)	40.9 (6.9)	40.6 (6.17)	41.1 (7.69)	42.1 (6.6)	0.849
HIV acquired by IVDU	85.4% (223)	85.1% (86)	83.7% (103)	91.9% (34)	0.556
Years since HCV infection	21 (8.4)	21.3 (8.35)	20.9 (8.25)	21.6 (7.8)	0.670
Prior AIDS	29.1% (76)	69.3% (70)	74.8% (92)	37.8% (14)	0.302
cART	84.7% (221)	82.2% (83)	87.8% (108)	81.1% (30)	0.410
Time on cART (years)	4.8 (5)	4.6 (4.9)	5 (4.8)	4.7 (5.1)	0.350
Current cART protocols					
Any NRTIs + any PI	24.5% (64)	25.7% (26)	25.2% (31)	18.9% (7)	0.691
Any NRTIs + PI + NNRTI	1.1% (3)	1% (1)	1.6% (2)	0% (0)	0.705
Any NRTIs + any NNRTI	51.3% (134)	50.5% (51)	51.2% (63)	54.1% (20)	0.933
Only NRTIs	7.3% (19)	5% (5)	9.8% (12)	5.4% (2)	0.346
Specific antiretroviral drugs					
Zidovudine	27.2% (71)	25.7% (26)	30.1% (37)	21.6% (8)	0.547
Stavudine	26.1% (68)	24.8% (25)	28.5% (35)	21.6% (8)	0.659
Didanosine	16.9% (44)	18.8% (19)	13.8% (17)	21.6% (8)	0.431
Tenofovir	26.8% (70)	28.7% (29)	22% (27)	37.8% (14)	0.138
Abacavir	16.1% (42)	9.9% (10)	21.1% (26)	16.2% (6)	0.075
Efavirenz	30.3% (79)	29.7% (30)	30.1% (37)	32.4% (12)	0.951
Ritonavir (r)	6.1% (16)	5.9% (6)	7.3% (9)	2.7% (1)	0.588
Lopinavir/r	12.3% (32)	10.9% (11)	12.2% (15)	16.2% (6)	0.700
Saquinavir	1.1% (3)	0% (0)	1.6% (2)	2.7% (1)	0.332
Fosamprenavir	2.3% (6)	3% (3)	2.4% (3)	0% (0)	0.582
HIV markers					
Nadir CD4+ T-cells (cells/μL)	204 (223)	210 (262)	195 (221)	210 (201)	0.157
Nadir CD4+ <200 cells/μL	49% (128)	46.5% (47)	52% (64)	45.9% (17)	0.658
CD4+ T cells/μL	465 (325)	460 (435)	472 (234)	456 (251)	0.313
CD4+ ≥500 cells/μL	43.5% (113)	55.4% (56)	57.4% (70)	43.2% (16)	0.959
HIV-RNA <50 copies/mL	76.9% (200)	75% (75)	78% (96)	78.4% (29)	0.844
HCV markers					
HCV-genotype 1/4	73.4% (185)	68.8% (66)	75% (90)	80.6% (29)	0.339
HCV-RNA ≥500,000 UI/ml	75.1% (187)	29.6% (29)	23.5% (27)	83.3% (30)	0.275
*IFNL3* rs12980275 (AA)	47.5% (122)	47.5% (48)	50% (61)	38.2% (13)	0.557

Categorical variables are expressed in percentage (absolute count).; continuous variables are expressed in median (interquartile range) *P*-values were estimated with Chi-square test for categorical variable and linear regression test for continuous variable. AIDS, acquired immunodeficiency syndrome; cART, combination antiretroviral therapy; HCV, hepatitis C virus; HCV-RNA, HCV plasma viral load; HIV, human immunodeficiency virus; HIV-RNA, HIV plasma viral load; IVDU, intravenous drug users; NNRTI, no nucleoside analog reverse-transcriptase inhibitors; NRTI, nucleoside analog reverse-transcriptase inhibitors; PI, protease inhibitors.

### *FTO* polymorphism frequencies

Allele frequencies for the rs9939609 polymorphism were 0.62 for T allele and 0.38 for A allele. Genotype frequencies were 0.39, 0.47 and 0.14 for TT, TA, AA genotypes, respectively. These frequencies in our dataset were in accordance with the data listed on the National Center for Biotechnology Information (NCBI) SNP database [32]. The rs9939609 SNP fulfilled the minimum allele frequency (MAF) >0.05 for all samples and displayed less than 5% of missing values. Furthermore, rs9939609 polymorphism was in HWE (*P* = 0.999).

### *FTO* polymorphism, metabolic disturbances and liver disease

Table 2 shows the relationship between the metabolic disturbances and the rs9939609 polymorphism under a model of recessive inheritance, which was the genetic model that best fit our data. Therefore, the risk conferred by homozygous for the minor A allele is increased r-fold in comparison to the rest (TT and AT genotypes).

**Table 2:** **Relationship between rs9939609 polymorphism**

**Outcomes**	**All patients**	**TT**	**AT**	**AA**	***P*** **-value** ^**(a)**^	**aAMR (95%CI)**	***P*** **-value** ^**(b)**^
Continuous variables							
BMI (kg/m^2^)	23.29 ± 0.25	22.69 ± 0.35	22.91 ± 0.32	24.29 ± 0.58	**0.016**	1.08 (1.03; 1.14)	**0.002**
HOMA-IR	3.24 ± 0.19	2.97 ± 0.29	2.75 ± 0.26	4.19 ± 0.47	**0.003**	1.32 (1.03; 1.69)	**0.027**
	**All patients**	**TT**	**AT**	**AA**	***P*** **-value** ^**(a)**^	**aOR (95%CI)**	***P*** **-value** ^**(b)**^
Categorical variables							
Overweight (BMI ≥27.5 kg/m^2^)	8.5% (22/260)	4% (4/100)	8.9% (11/123)	19.4% (7/36)	**0.011**	3.46 (1.17; 10.21)	**0.024**
HOMA-IR ≥2.5	41.4% (108/261)	38.6% (39/101)	39% (48/123)	56.8% (21/37)	**0.040**	2.09 (1.02; 4.32)	**0.045**
Significant fibrosis (F ≥ 2)	48.6% (102/210)	48.8% (41/84)	43.3% (42/97)	65.5% (19/29)	**0.049**	2.34 (1.02; 5.36)	**0.045**
Steatosis (>10% fatty hepatocytes)	56.9% (115/202)	51.9% (41/79)	57% (53/93)	70% (21/30)	0.117	3.65 (1.29;10.36)	**0.015**

^a^ *P*-values were calculated by Chi-square tests for categorical variables and generalized linear models (GLM) with normal distribution (log-link) for continuous variables; ^b^ *P*-values were calculated by GLM adjusted by the most important clinical and epidemiological characteristics (see Statistical analysis section). Metabolic disturbances and liver disease in HIV/HCV-coinfected patients. Categorical variables are expressed in percentage (absolute count) and continuous variables are expressed in estimated marginal mean ± standard error of the mean. Statistically significant differences are shown in bold. 95%CI, 95% confidence interval; aAMR, adjusted arithmetic mean ratio; aOR, adjusted odds ratio; BMI, body mass index; HCV, hepatitis C virus; HIV, human immunodeficiency virus; HOMA-IR, homeostatic model assessment-insulin resistance.

Patients with the rs9939609 AA genotype had higher values of BMI (*P* = 0.016) and HOMA-IR (*P* = 0.003) than patients with the rs9939609 AT/TT genotype. Also, the rs9939609 AA genotype had a higher percentage of patients with BMI ≥27.5 kg/m^2^ (*P* = 0.011), HOMA-IR ≥2.5 (*P* = 0.040) and significant fibrosis (*P* = 0.049) than rs9939609 AT/TT. When the multivariate regression analyses were performed, we found that the rs9939609 AA genotype was associated with higher values of BMI (adjusted AMR (aAMR) = 1.08; *P* = 0.002) and HOMA-IR values (aAMR = 1.32; *P* = 0.027), and higher likelihoods of achieving values of BMI ≥27.5 kg/m^2^ (adjusted OR (aOR) = 3.46; *P* = 0.024), HOMA-IR ≥2.5 (aOR = 2.09; *P* = 0.045), significant fibrosis (aOR = 2.34; *P* = 0.045) and steatosis (aOR = 3.65; *P* = 0.015).

Additional file 1: Tables S1 and S2 show the analyses stratified by HCV-GT1 and HCV-GT3, respectively. For HCV-GT1 patients, the rs9939609 AA genotype was only linked to HOMA-IR (aAMR = 1.52; *P* = 0.017). With regard to HCV-GT3 patients, the rs9939609 AA genotype was associated with elevated BMI values (aAMR = 1.18; *P* = 0.004).

### *FTO* polymorphism and virologic response to HCV treatment

Table 3 shows the relationship between the rs9939609 polymorphism and the virologic responses to HCV treatment under an additive model of inheritance. Therefore, the risk conferred by an allele is increased r-fold for heterozygotes and 2r-fold for homozygotes with two copies of a specific allele.

**Table 3:** **Relationship between rs9939609 polymorphism and virologic responses to HCV treatment in HIV/HCV-coinfected patients according to HCV genotypes**

**HCV genotype**	**All patients**	**TT**	**AT**	**AA**	***P*** **-value** ^**(a)**^	**aOR (95%CI)**	***P*** **-value** ^**(b)**^
**All patients**	55.1% (98/178)	64.3% (45/70)	51.8% (43/83)	40.0% (10/25)	**0.026**	0.58 (0.34; 0.99)	**0.044**
**GT2/3 patients**	85.5% (47/55)	92.0% (23/25)	83.3% (20/24)	66.7% (4/6)	0.125	0.43 (0.14; 1.31)	0.138
**GT1/4 patients**	40.7% (48/118)	48.8% (20/41)	37.9% (22/58)	31.5% (6/19)	0.171	0.59 (0.31; 1.12)	0.105

^a^ *P*-values were calculated by linear-by-linear association Chi-squared test; ^b^ *P*-values were calculated by multivariate generalized linear models (GLM) adjusted by the most important clinical and epidemiological characteristics (see Statistical analysis section). Statistically significant differences are shown in bold. 95%CI, 95% confidence interval; aOR, adjusted odds ratio; GT1/4, HCV genotype 1/4; GT2/3, HCV genotype 2/3; HCV, hepatitis C virus; HIV, human immunodeficiency virus; SVR, sustained virologic response.

We analyzed 178 of 261 patients who were treated with pegIFNα/RBV. The number of patients who failed to complete HCV therapy was 16 (12 adverse events and 4 abandonments) and 162 patients had a full course of HCV therapy. In an intention-to-treat analysis, the SVR rate was 55.1%, which decreased with each minor A allele at rs9939609 (*P* = 0.026). Additionally, the multivariate analysis revealed similar results, showing that rs9939609 A allele decreased the likelihood of achieving SVR (aOR = 0.58; *P* = 0.044).

**Figure 2:**
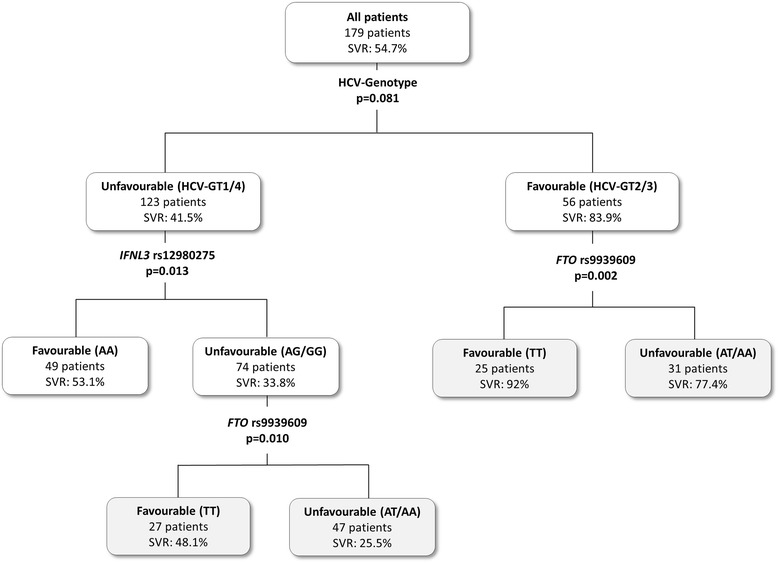
**Flowchart of the decision tree for sustained virologic responses (SVR) in HIV patients coinfected with HCV.** Nodes have been stratified by HCV-GT (1/4 versus 2/3), IFNL3 rs12980275 (AA versus AG/GG) and FTO rs9939609 (TT versus AT/AA) polymorphisms. HCV-GT, hepatitis C virus genotype.

A decision tree was also performed using both rs12980275 and rs9939609 polymorphisms (Figure 2). For GT1/4 patients, the SVR rate decreased from 41.5% to 33.8% in patients with the rs12980275 AG/GG genotype and then to 25.5% in patients with the rs9939609 AT/AA genotype while it increased to 48.1% in patients with the rs9939609 TT genotype. For GT2/3 patients, the SVR rate decreased from 83.9% to 77.4% in patients with the rs9939609 AT/AA genotype while it increased to 92.0% in patients with the rs9939609 TT genotype. The overall percentage of patients correctly classified (accuracy) was 68.2% and the AUROC of this decision tree was 0.766 (95%CI = 0.696; 0.835. *P* <0.001). Furthermore, this analysis was replicated considering the HCV-GT1 and HCV-GT3 separately (Additional file 1: Table S1 and Figure S1), finding similar results to those previously described (Figure 2).

## Discussion

The association between the *FTO* rs9939609 polymorphism and metabolic factors is well documented in the general population [19,22-24]. However, to the best of our knowledge, this is the first description of the relation between the *FTO* rs9939609 polymorphism and metabolic disturbances in HIV/HCV-coinfected patients. In this study, we showed that patients with an rs9939609 AA genotype had increased odds of obesity, insulin resistance, significant fibrosis and liver steatosis; and the presence of the risk A allele (both AA and AT genotypes) was associated with a lower response rate to pegIFNα/ribavirin therapy. This effect does not appear to be dependent on the HCV genotype, although we could not confirm this with certainty due to a lack of statistical power when we performed the analysis stratified by HCV genotype.

Obesity is a complex metabolic disturbance in which genetic and environmental factors may interact to produce homeostatic abnormalities [21], but in addition, both the HIV/HCV coinfection and the cART also influence the development of other comorbidities, such as insulin resistance and steatosis [7]. Recent meta-analyses have reliably established that *FTO* rs9939609 is robustly associated with increased BMI and adiposity across different ages and populations [22,33]. Evidence to date suggests that the association between rs9939609 and BMI is predominantly driven by an increased energy intake, and that patients with the rs9939609 AA genotype exhibit overall increased *ad libitum* food-intake and impaired satiety [34-37]. In our study, subjects homozygous for the ‘obesity-risk’ A allele also had an increased risk for overweight/obesity (both continuous BMI values and BMI ≥27.5 kg/m^2^) compared with carriers of the low-risk T allele. Nevertheless, we must not discount the fact that our patients had a relatively low BMI (only 6.5% of patients were overweight), possibly due to the fact that around 85% of them were IDUs. The HIV infection and chronic drug abuse both compromise the nutritional status of patients despite major advances in HIV treatment [38], allowing that HIV-positive IDUs had lower BMIs.

The relationship between HCV and obesity is clinically relevant due to the potential synergism on liver disease severity and the multifaceted interactions between HCV and glucose metabolism [26]. HCV infection is able to perturb glucose homeostasis through several direct and indirect mechanisms, leading to both hepatic and extrahepatic insulin resistance [26], which accelerates liver disease progression (development of steatosis, fibrosis and hepatocellular carcinoma), reduces response to HCV antivirals, and increases the risk of developing type 2 diabetes mellitus [26]. However, the mechanisms by which CHC leads to insulin resistance are not fully understood. There is growing evidence that DNA polymorphisms may contribute to differences in complex disease traits between individuals. In this setting, many studies have investigated the relationship of the *FTO* rs9939609 polymorphism with insulin resistance and type 2 diabetes mellitus in the general population (HIV and HCV seronegative subjects) [19,24,39-41]. According with this, our study revealed that coinfected patients carrying the rs9939609 AA genotype had higher HOMA-IR values and a frequency of HOMA-IR ≥2.5 than patients carrying the rs9939609 AT/TT genotype. However, our data revealed that the *FTO* rs9939609 polymorphism was not associated with HOMA-IR ≥3.0 and HOMA-IR ≥3.8 (data not shown), possibly due to the limited sample size, since a near-significant trend was observed. Moreover, this lack of association could be due to the possible distortive effect of direct and indirect factors related to both HIV and HCV infections, and cART [7].

Liver steatosis is a frequent finding in CHC (≥40% cases). In HCV infection with GT3, viral factors are implicated in the development of steatosis via activation of *de novo* lipogenesis, while for HCV infection with GT1 or GT4, metabolic host factors, such as obesity and insulin resistance, provide an increased risk of steatosis [26]. Additionally, oxidative damage induced by the HCV core protein may simultaneously induce steatosis and impair insulin signaling in the hepatocyte [26]. The *FTO* gene is highly expressed in the hypothalamus and pancreatic islets, and it is found in other tissues such as adipose tissue, liver, and skeletal muscle. The *FTO* over-expression level seems to be related to subcutaneous fat accumulation [42], obesity [43] and oxidative stress and lipid deposition in the liver [44]. Furthermore, the rs9939609 AA genotype exhibits increased *FTO* expression compared with the TT genotype [35]. In our study, the rs9939609 AA genotype was related to higher likelihoods of steatosis, a finding that is consistent with the relationship found between rs9939609, obesity and insulin resistance in these same patients. This fact could have important clinical implications because persistent fatty liver disease may be a problem for patients even with successful HCV clearance [45,46].

Our analysis also shows that the rs9939609 polymorphism was associated with increased odds of significant liver fibrosis. Considering that the development of liver fibrosis is strongly associated with overweight/obesity, insulin resistance and steatosis [7,47,48], we think that the association between the *FTO* rs9939609 polymorphism and liver fibrosis might be mediated by the metabolic disorders related to CHC.

Considering all the factors discussed above, it could be possible that the *FTO* rs9939609 polymorphism might be related to response to HCV therapy through regulating obesity, insulin resistance and liver steatosis in CHC patients [26]. In our study, the rs9939609 A risk-allele showed an inverse significant association with SVR. These associations remained after adjusting for the most important predictive factors related to HCV treatment response, such as *IFNL3* genotype, HOMA-IR, HCV genotype, HCV-RNA viral load, and so on [13]. However, when patients were stratified by HCV genotype, the trends were maintained but were not statistically significant, possibly due to the limited sample size. In any case, we did not observe an association that could be dependent on HCV genotype. Moreover, we made an algorithm based on the genotypes of HCV, *IFNL3* and *FTO*, finding that the *FTO* genotype was able to improve the classification into responder and non-responder to pegIFNα/ribavirin therapy for difficult-to-treat patients (GT1/4) with an unfavourable *IFNL3* genotype (rs12980275 AG/GG). Moreover, the classification of GT2/3 patients also improved without the assistance of the *IFNL3* genotype. In this case, the rs12980275 genotype was not included in the GT2/3 decision tree due to two main reasons: 1) For a decision tree analysis, the sample size in the GT2/3 group (56 patients) was quite limited to obtain valid results; and thus, the second node was pruned because it had fewer than 10 patients. 2) IL28B SNPs are not been useless for predicting HCV therapy outcome in HIV/HCV-coinfected patients infected with HCV-GT2/3 [49]. This fact is may be due to the high SVR rate found in these IFN-sensitive genotypes, where a larger sample size would be required to establish statistical differences [50]. Thus, the *FTO* rs9939609 polymorphism might provide a new clinical value in patients who have no access to newer DAAs treatment.

To date, many articles have assessed the influence of *IL28B* polymorphisms on SVR in CHC patients, rs12979860, rs8099917, and rs12980275 being the most studied [51]. These *IL28B* polymorphisms are being used as predictive markers of response to pegIFNα/RBV therapy in clinical practice, especially in patients with HCV genotypes 1 and 4 [13]. Although rs12979860 is more likely to be correlated with SVR in the European white population, we have recently shown a strong association of rs12980275 and rs8099917 with SVR in HCV/HIV-coinfected patients [52]. In the current study, we analyzed rs12980275, which is also in high linkage disequilibrium with rs8099917 and rs12979860 in the European population [50]. In addition, rs12980275 has been less studied than rs12979860 in European white populations, and, therefore, additional results involving rs12980275 would be of interest.

At present, the new DAAs have achieved a very high response rate [17]. This fact might obscure the influence on treatment efficacy of *IFNL3* polymorphisms and other SNPs, such as the *FTO* rs9939609 polymorphism. However, some authors have still suggested that the *IFNL3* genotype plays a key role for certain IFN-free regimens since several clinical trials have revealed an association between *IFNL3* polymorphisms and treatment efficacy [53]. Moreover, the role of tools for making pre-treatment decisions may still be relevant since DAAs are more expensive and carry a higher risk for side effects, while decision-making based on *IFNL3* polymorphisms and other SNPs might permit non-DAA-based treatment algorithms. Finally, it should also be borne in mind that few data are available in HIV/HCV coinfected patients about the interaction of IFN-free regimens and metabolic disturbances, where FTO is a cornerstone. Thus, further analysis will be needed to determine whether *FTO* rs9939609 polymorphism could provide additional information to select patients with better or worse response to HCV treatment.

There are some issues that have to be considered for a correct interpretation of our data:

Firstly, this report has a cross-sectional design (analysis of metabolic disturbances) and a retrospective design (analysis of virological response to HCV therapy), both with a relatively small number of patients, which could limit the achievement of significant values between the rs9939609 polymorphism and the outcome variables (for example: BMI ≥25 kg/m^2^, HOMA-IR ≥3.0, HOMA-IR ≥3.8, HCV-therapy response according to HCV genotype, and so on). In addition, our cohort had a population with mixed HCV genotypes (1, 2, 3 and 4), which complicates the interpretation of the data since, for example, GT1 and GT3 did not have exactly the same pathophysiology and response to HCV treatment.

Secondly, metabolic disturbances are caused by several interacting genetic and environmental determinants, making it complicated to find the true individual effects of each disease-associated factor. In this regard, we did not have data on some extra factors that may have an influence on lipid levels and insulin resistance, such as exercise habits, diet, lipodystrophy, and alcohol intake.

Thirdly, the patients selected for our study were patients who met a set of criteria for starting HCV treatment (for example, no alcohol abuse, high CD4 cell counts, controlled HIV replication and good treatment adherence), and it is possible that this may have introduced a selection bias. Furthermore, HCV therapy regimens were not identical since they varied in some characteristics, such as pegIFNα 2a or 2b and likely ribavirin dose. Instead, each physician administered the appropriate HCV therapy regimen according to his/her criteria and by following local and/or international guidelines.

Fourthly, we did not study any cohort of HCV-monoinfected patients in order to evaluate the influence of the *FTO* rs9939609 polymorphism on CHC without the presence of HIV infection. In addition, we did not study any cohort of HIV-monoinfected patients in order to evaluate the influence of the *FTO* rs9939609 polymorphism on the development of metabolic disturbances with the presence of HIV infection and cART. Moreover, since the study was carried out entirely in white Europeans, and the frequency of these alleles differs among different ethnicities, it would be necessary to perform an independent replication of this study for different ethnic groups.

Fifthly, we evaluated several outcome variables and could raise the need to adjust the ‘*P*-value’. However, we think that it is not necessary to adjust the ‘P-value’ after multiple tests on clinical-orientated studies [54,55], because: 1) the outcome variables cannot be considered completely independent; 2) there was a hypothesis supported by theory and we were not doing a random search of a meaningful result; and 3) our results had a clear interpretation. Thus, our results, which always pointed in the same direction, should not be ruled out. However, we should not overlook the level of uncertainty of these data.

## Conclusions

Our study shows the first evidence that HCV/HIV-coinfected patients carrying the unfavourable A allele of the *FTO* rs9939609 polymorphism had higher odds of metabolic disturbances and lower likelihoods of achieving a successful virologic response to HCV therapy. Further analyses are needed to determine the potential use of the rs9939609 polymorphism as a predictive marker of metabolic disturbances and HCV therapy response.

## Additional file

Additional file 1: Table S1. Relationship between rs9939609 polymorphism, metabolic disturbances and liver disease in HIV patients coinfected with HCV-GT1. **Table S2.** Relationship between rs9939609 polymorphism, metabolic disturbances and liver disease in HIV patients coinfected with HCV-GT3. **Table S3.** Relationship between rs9939609 polymorphism and virologic responses to HCV treatment in HIV/HCV-coinfected patients according to HCV genotypes 1 and 3. **Figure S1.** Flowchart of the decision tree for sustained virologic responses (SVR) in HIV patients coinfected with HCV. Nodes have been stratified by HCV-GT (1 versus 3), IFNL3 rs12980275 (AA versus AG/GG) and FTO rs9939609 (TT versus AT/AA) polymorphisms.

## Abbreviations

95%CI: 95% confidence interval
aAMR: adjusted arithmetic mean ratio
AIDS: acquired immunodeficiency syndrome
aOR: adjusted odds ratio
BMI: body mass index
cART: combination antiretroviral therapy
CHC: chronic hepatitis C
DAAs: direct-acting antivirals
GLM: generalized linear model
GT1/4: HCV genotype 1/4
GT2/3: HCV genotype 2/3
GWAS: genome-wide association study
HCV: hepatitis C virus
HCV-RNA: HCV plasma viral load
HIV: human immunodeficiency virus
HIV-RNA: HIV plasma viral load
HOMA: homeostatic model assessment
HWE: Hardy-Weinberg equilibrium
IFN: interferon
IR: insulin resistance
IVDU: intravenous drug users
NNRTI: no nucleoside analog reverse-transcriptase inhibitors
NRTI: nucleoside analog reverse-transcriptase inhibitors
pegIFNα/RBV: pegylated-interferon-alpha plus ribavirin
PI: protease inhibitors
SNP: single nucleotide polymorphism
SVR: sustained virologic response
