# Supplementary material for: FTO rs9939609 polymorphism is associated with metabolic disturbances and response to HCV therapy in HIV/HCV-coinfected patients
Source: BMC Med. 2014 Nov 3;12:198. doi: 10.1186/s12916-014-0198-y (PMC4224698; doi:10.1186/s12916-014-0198-y)

**Additional file 1: Table S1.** Relationship between rs9939609 polymorphism, metabolic disturbances and liver disease in HIV patients coinfected with HCV-GT1.

| **Outcomes** | **All patients** | **TT** | **AT** | **AA** | **p-value ^(a)^** | **aAMR (95%CI)** | **p-value ^(b)^** |
| --- | --- | --- | --- | --- | --- | --- | --- |
| **Continuous variables** |  |  |  |  |  |  |  |
| **BMI (kg/m^2^)** | 23.2 ± 0.37 | 23.04 ± 0.44 | 22.6 ± 0.40 | 23.6 ± 0.68 | 0.258 | 1.03 (0.97; 1.09) | 0.317 |
| **HOMA-IR** | 3.45 ± 0.29 | 3.09 ± 0.38 | 2.73 ± 0.35 | 4.13 ± 0.60 | **0.036** | 1.52 (1.08; 2.13) | **0.017** |
|  |  |  |  |  |  |  |  |
|  | **All patients** | **TT** | **AT** | **AA** | **p-value ^(a)^** | **aOR (95%CI)** | **p-value ^(b)^** |
| **Categorical variables** |  |  |  |  |  |  |  |
| **Overweight (BMI ≥27.5 kg/m^2^)** | 6.3% (9/142) | 3.6% (2/55) | 6.2% (4/65) | 13.6% (3/22) | 0.145 | 1.78 (0.27; 11.70) | 0.550 |
| **HOMA-IR ≥2.5** | 41.5% (59/142) | 43.6% (24/55) | 38.5% (25/65) | 45.5% (10/22) | 0.686 | 1.38 (0.42; 4.56) | 0.595 |
| **Significant fibrosis (F≥2)** | 48.7% (57/117) | 52.1% (25/48) | 47.2% (25/53) | 43.8% (7/16) | 0.669 | 0.61 (0.19; 2.01) | 0.415 |
| **Steatosis (>10% fatty hepatocytes)** | 53.2% (58/109) | 45.2% (19/42) | 54.2% (26/48) | 68.4% (13/19) | 0.114 | 2.91 (0.86; 9.93) | 0.087 |

Categorical variables are expressed in percentage (absolute count) and continuous variables are expressed in estimated marginal mean ± standard error of mean. Statistically significant differences are shown in bold. (a), P-values were calculated by Chi-square tests for categorical variables and generalized linear models (GLM) with normal distribution (log-link) for continuous variables. (b), P-values were calculated by GLM adjusted by the most important clinical and epidemiological characteristics (see **statistical analysis** section). 95%CI, 95% of confidence interval; aAMR, adjusted arithmetic mean ratio; aOR, adjusted odds ratio; BMI, body mass index; HCV, hepatitis C virus; HIV, human immunodeficiency virus; HOMA-IR, homeostatic model assessment-insulin resistance.

**Additional file 1: Table S2.** Relationship between rs9939609 polymorphism, metabolic disturbances and liver disease in HIV patients coinfected with HCV-GT3.

| **Outcomes** | **All patients** | **TT** | **AT** | **AA** | **p-value ^(a)^** | **aAMR (95%CI)** | **p-value ^(b)^** |
| --- | --- | --- | --- | --- | --- | --- | --- |
| **Continuous variables** |  |  |  |  |  |  |  |
| **BMI (kg/m^2^)** | 23.4 ± 0.70 | 22.1 ± 0.61 | 23.2 ± 0.65 | 24.2 ± 1.37 | 0.251 | 1.18 (1.06; 1.33) | **0.004** |
| **HOMA-IR** | 3.34 ± 0.59 | 3.34 ± 0.54 | 2.57 ± 0.58 | 3.74 ± 1.22 | 0.521 | 1.32 (0.67; 2.59) | 0.418 |
|  |  |  |  |  |  |  |  |
|  | **All patients** | **TT** | **AT** | **AA** | **p-value ^(a)^** | **aOR (95%CI)** | **p-value ^(b)^** |
| **Categorical variables** |  |  |  |  |  |  |  |
| **BMI ≥27.5 kg/m^2^** | 6.5% (4/62) | 6.7% (2/30) | 3.8% (1/26) | 16.7% (1/6) | 0.342 | 2.05 (0.01; 350.48) | 0.784 |
| **HOMA-IR ≥2.5** | 38.1% (24/63) | 26.7% (11/30) | 30.8% (8/26) | 71.4% (5/7) | 0.095 | 6.89 (0.54; 88.26) | 0.138 |
| **Significant fibrosis (F≥2)** | 62.5% (30/48) | 50% (11/22) | 63.2% (12/19) | 100% (7/7) | **0.036** | - | 0.999 |
| **Steatosis (>10% fatty hepatocytes)** | 70% (35/50) | 66.7% (16/24) | 63.2 (12/19) | 100% (7/7) | 0.087 | - | 0.999 |

Categorical variables are expressed in percentage (absolute count) and continuous variables are expressed in estimated marginal mean ± standard error of mean. Statistically significant differences are shown in bold. (a), P-values were calculated by Chi-square tests for categorical variables and generalized linear models (GLM) with normal distribution (log-link) for continuous variables. (b), P-values were calculated by GLM adjusted by the most important clinical and epidemiological characteristics (see **statistical analysis** section). 95%CI, 95% of confidence interval; aAMR, adjusted arithmetic mean ratio; aOR, adjusted odds ratio; BMI, body mass index; HCV, hepatitis C virus; HIV, human immunodeficiency virus; HOMA-IR, homeostatic model assessment-insulin resistance.

**Additional file 1: Table S3**. Relationship between rs9939609 polymorphism and virologic responses to HCV treatment in HIV/HCV-coinfected patients according to HCV genotypes 1 and 3.

| **HCV genotype** | **All patients** | **TT** | **AT** | **AA** | **p-value ^(a)^** | **aOR (95%CI)** | **p-value ^(b)^** |
| --- | --- | --- | --- | --- | --- | --- | --- |
| **All patients** | 55.1% (98/178) | 64.3% (45/70) | 51.8% (43/83) | 40.0% (10/25) | **0.026** | 0.58 (0.34; 0.99) | **0.044** |
| **GT3 patients** | 84.6% (44/52) | 91.7% (22/24) | 81.8% (18/22) | 66.7% (4/6) | 0.128 | 0.44 (0.14; 1.32) | 0.141 |
| **GT1 patients** | 45.1% (41/91) | 50% (17/34) | 45.5% (20/44) | 30.8% (4/13) | 0.276 | 0.64 (0.32; 1.27) | 0.202 |

Statistically significant differences are shown in bold. (a), P-values were calculated by linear-by-linear association Chi-squared test; (b), P-values were calculated by multivariate generalized linear models (GLM) adjusted by the most important clinical and epidemiological characteristics (see **statistical analysis** section). 95%CI, 95% of confidence interval; aOR, adjusted odds ratio; GT1, HCV genotype 1; GT3, HCV genotype 3; HCV, hepatitis C virus; HIV, human immunodeficiency virus; SVR, sustained virologic response.

**Additional file 1: Figure S1.** Flowchart of the decision tree for sustained virologic responses (SVR) in HIV patients coinfected with HCV. Nodes have been stratified by HCV-GT (1 vs 3), IFNL3 rs12980275 (AA vs AG/GG) and FTO rs9939609 (TT vs AT/AA) polymorphisms. HCV-GT, hepatitis C virus genotype.


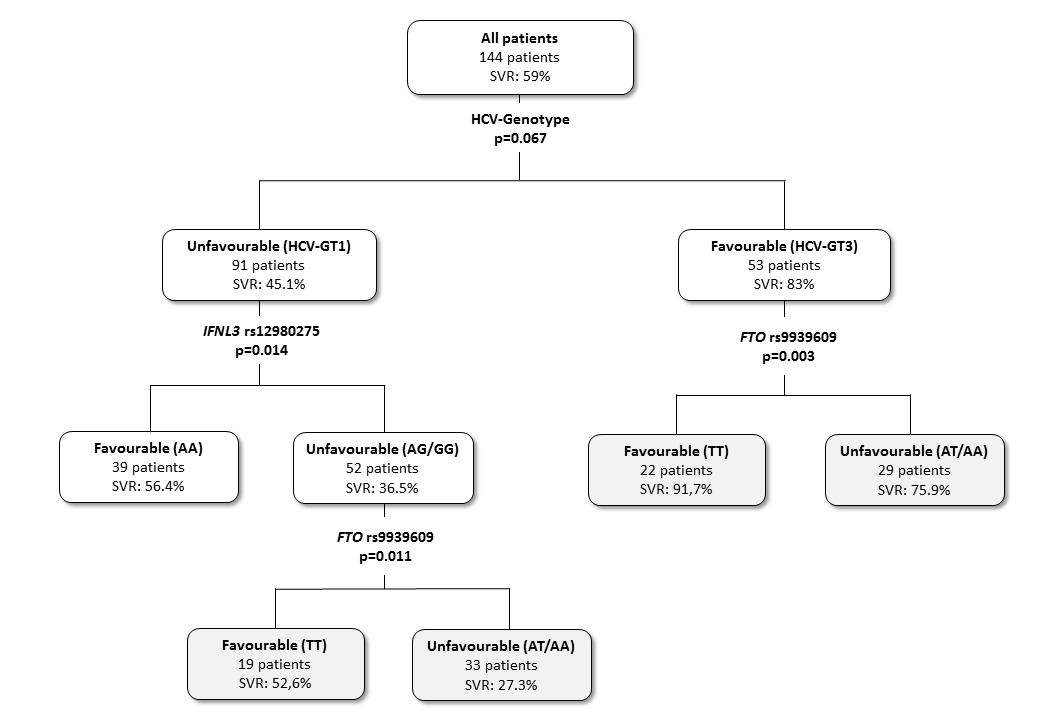

Supplement: Additional file 1: Table S1. — Relationship between rs9939609 polymorphism, metabolic disturbances and liver disease in HIV patients coinfected with HCV-GT1. Table S2. Relationship between rs9939609 polymorphism, metabolic disturbances and liver disease in HIV patients coinfected with HCV-GT3. Table S3. Relationship between rs9939609 polymorphism and virologic responses to HCV treatment in HIV/HCV-coinfected patients according to HCV genotypes 1 and 3. Figure S1. Flowchart of the decision tree for sustained virologic responses (SVR) in HIV patients coinfected with HCV. Nodes have been stratified by HCV-GT (1 versus 3), IFNL3 rs12980275 (AA versus AG/GG) and FTO rs9939609 (TT versus AT/AA) polymorphisms. [file 12916_2014_198_MOESM1_ESM.doc]
